# Enhancement of Rabbit Meat Functionality by Replacing Traditional Feed Raw Materials with Alternative and More Sustainable Freshwater *Cladophora glomerata* Macroalgal Biomass in Their Diets

**DOI:** 10.3390/foods12040744

**Published:** 2023-02-08

**Authors:** Monika Nutautaitė, Asta Racevičiūtė-Stupelienė, Saulius Bliznikas, Vilma Vilienė

**Affiliations:** 1 Institute of Animal Rearing Technologies, Veterinary Academy, Lithuanian University of Health Sciences, LT-47181 Kaunas, Lithuania; 2 Institute of Animal Science, Lithuanian University of Health Sciences, LT-82317 Baisogala, Lithuania

**Keywords:** amino acids, fatty acids, lipid stability, functional food, bioactive compounds, alternative feedstuff, macroalgae

## Abstract

Today’s challenges in the animal husbandry sector, with customers’ demand for more beneficial products, encourage the development of strategies that not only provide more sustainable production from the field to the table but also ensure final product functionality. Thus, the current research was aimed at replacing some traditional feed raw materials in rabbit diets with *C. glomerata* biomass to improve the functionality of meat. For this purpose, thirty weaned (52-d-old) Californian rabbits were assigned to 3 dietary treatments: standard compound diet (SCD), SCD + 4% *C. glomerata* (CG4), and SCD + 8% *C. glomerata* (CG8). At the end of the feeding trial, 122-d-old rabbits were slaughtered, *longissimus dorsi* (LD) and hind leg (HL) muscles were dissected post-mortem, and moisture, protein, and lipid profiles were determined. Results revealed that CG4 treatment can increase protein (22.17 g/kg), total (192.16 g/kg) and essential (threonine, valine, methionine, lysine, and isoleucine) amino acid levels in rabbit muscles. Both inclusions gradually reduced fat accumulation in muscles (CG8 < CG4 < SCD) but improved the lipid profile’s nutritional value by decreasing saturated fatty acids (SFA) and monounsaturated fatty acids (MUFA) and increasing polyunsaturated fatty acids (PUFA). As the dose of *C. glomerata* increased, the level of lipid oxidation decreased. Biomass supplementation enhanced PUFA/SFA and h/H levels while decreasing thrombogenicity index (TI) and atherogenic index (AI) levels in rabbit muscles, potentially contributing to the prevention of heart disease. Overall, dietary supplementation with *C. glomerata* biomass may be a more beneficial and sustainable nutritional approach to functionally enhancing rabbit meat.

## 1. Introduction

A long-term strategy for developing intensive but sustainable livestock farming is required, as a rapidly growing population directly correlates with steadily increasing demand for high-quality animal products. Considering the economic benefits and today’s challenges, to at least partially replace traditional feed additives that are predicted to be in short supply, novel feed materials are being sought [1]. It is necessary not only to search for but also to analyse the potential uses of alternative feed materials to develop effective technological preparation and volarization methods, as well as to ensure high quality and safety, including sustainability, antinutritional factors, and potential toxicity, ultimately creating functional feed and a functional final product. Algae, for instance, are cultivated and used for nutritional or other purposes all over the world, and their biomass is recognised as a source of essential vitamins, minerals, proteins, polyunsaturated fatty acids, and antioxidants [2,3]. The great potential of algae comes from the fact that, compared to conventional agricultural crops, they can be grown in places where other plants cannot grow, and their biomass is produced extremely quickly [4]. Due to the more efficient use of solar energy, algae can produce biomass enriched with bioactive compounds that can be used to improve the nutritional value of food and feed [5]. Algae is already successfully used as animal feed or as a functional feed additive in many parts of the globe [6,7,8,9]; and in the European Union it can be used as feed material according to Commission Regulation (EU) No. 68/2013 of 16 January 2013, regarding the catalogue of feed materials.

Specifically, *Cladophora* species, whether marine or freshwater, are distinguished as ecologically and economically important macroalgae. These species perform key ecosystem functions, and their biomass is used for a variety of purposes, such as soil improvement additives, fertilizers, plant growth stimulants, food and animal feed, pharmaceuticals, cosmetics, wastewater treatment, and renewable biofuels [10,11]. The green macroalgae *Cladophora glomerata* (*C. glomerata*) thrives and forms large communities in nutrient-rich bodies of water, especially slow-flowing rivers [12]. However, the blooms cause a reduction in biodiversity since the biomass assemblage consists mainly of only one species of green algae, reducing the recreational value of water bodies and creating harmful ecological and economic impacts. Frequently, due to its relatively high protein content, *C. glomerata* is recommended to be integrated into both human and animal diets [13]. Both the food and feed industries utilise this biomass as a raw material that contains few calories but many nutrients, vitamins, and fibres [14]; specifically, *C. glomerata* is characterised by a rich spectrum of bioactive components, which is reflected in the chemical composition, especially in the profiles of amino and fatty acids, phenolic compounds [3,8,14,15,16]. Thus, when viewed through the lens of today’s animal husbandry problems (general concept of sustainability, lack of feed materials, and greenhouse gas emissions), the biomass of *C. glomerata* macroalgae would be eligible for use as a feed ingredient. Thus, harvesting excess biomass from water bodies and incorporating it into feed production would adapt waste as a raw material, resulting in a more sustainable production chain.

According to recent research, *Cladophora* species have been commonly used to supplement aquaculture diets [17,18,19]. Furthermore, the incorporation of algae into animal feed has been demonstrated in experiments to help reduce intestinal dysbiosis in pigs and could potentially be an alternative in rabbit farming [20]. Additionally, algae integration in rabbit diets has the potential to become a commercial marketing strategy for attracting new consumers who are conscious of environmental sustainability and seeking innovative, high-quality foods. In this regard, it has already been established that supplementing animals’ feed with algae directly increases meat quality [17,19,21,22]. Moreover, there is limited research on the effects of *Cladophora* biomass on rabbits, and the demand for rabbit meat is expanding as the concept of healthy nutrition among consumers becomes more common [23]. Meat and its derivatives may be termed functional foods since they contain various beneficial components, and to create a more functional production, various strategies for combining qualitative or quantitative adjustments into meat occur [24]. However, functional feed must first be established in order to produce even more functional rabbit meat. Alternative feeds formulated with *C. glomerata* biomass, despite its higher fibre content, can be adapted to the diet of rabbits, considering their unique digestive physiology (caecotrophy) [25]. In this way, rabbits could achieve high productivity results and health status by eating high-fibre feed and, at the same time, produce functional meat production. Thus, the goal of this study was to replace some traditional feed raw materials in rabbit diets with different dosages of *C. glomerata* biomass to improve meat functionality and determine different muscle protein and lipid profiles.

## 2. Materials and Methods

### 2.1. Animal and Experimental Treatments

The research was conducted at a local rabbit breeding farm where animals were farmed indoors in individual cages (34 × 34 × 61 cm; 1 rabbit per cage) and had free access to individual nipple drinkers with clean drinking water and feed bowls to ensure optimal health conditions and performance. The heating system in the building maintained a temperature of 19 ± 2 °C. Housing standards were in accordance with Council Directive 98/58/EC of 20 July 1998, concerning the protection of animals kept for farming purposes.

The trial was carried out with thirty weaned (52-d-old) Californian breed male rabbits. Selected by similar weight, rabbits were randomly assigned to 3 dietary treatments (*n* = 10 rabbits/diet) and were fed twice a day with a standard compound diet (SCD), SCD supplemented with 4% freshwater *C. glomerata* biomass (CG4), and SCD supplemented with 8% *C. glomerata* biomass (CG8). The standard compound diet was formulated and analysed to cover the nutrient requirements of growing rabbits, including vitamins and minerals (Table 1), as recommended by the National Research Council [26].

**Figure 1:**
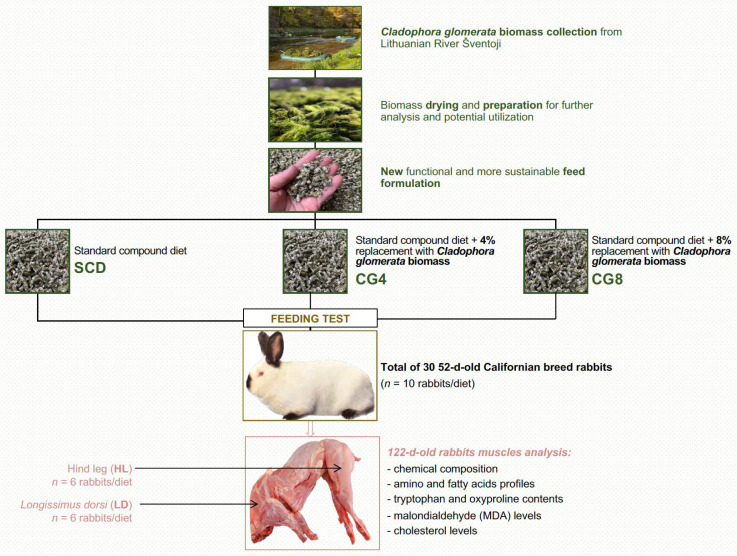
Protocol structure from the Šventoji River to rabbit muscle analysis.

Throughout the study, all the groups received the rations twice a day with an *ad libitum* access to the feed, supplied in the form of pellets. The biomass used in the production of feed was collected from the Šventoji River in Lithuania. Previously examined and described parameters of biomass chemical indicators, nutritional value, and antioxidant activity are published in previous studies [15,16,27]. The protocol structure from river to muscle analysis is presented in Figure 1.

### 2.2. Samples Collection

At the end of the feeding trial (122-d-old), 18 rabbits (*n* = 6 rabbits/diet) were randomly selected, weighed, starved overnight, and then euthanized in accordance with normal farming practice. The slaughter was carried out at a rabbit farm slaughterhouse in line with the established procedures which comply with the law of the Republic of Lithuania (Order No B1-866 of 31 October 2012 of the Director of the State Food and Veterinary Service on the approval of requirements for the keeping, care, and use of animals for scientific and educational purposes).

The carcasses of rabbits were prepared as reported by Blasco and Ouhayoun [28] and chilled at 4 °C for 24 h in a ventilated room. From the reference carcasses, the *Longissimus dorsi* (LD) and hind leg (HL) muscles were separated. The dissection procedures of warm and chilled carcasses followed the World Rabbit Science Association (WRSA) recommendations [28]. A total of 36 rabbit muscle samples were collected 24 h post-mortem, minced, and stored at −80 °C (as fresh meat; LD0 and HL0 (*n* = 6 LD/diet; *n* = 6 HL/diet)) and at −18 °C for 3 months (as stored meat; and LD3 and HL3 (*n* = 6 LD/diet; *n* = 6 HL/diet)) for later malondialdehyde (MDA) analysis. Other meat quality traits (chemical composition, amino and fatty acids profiles, and cholesterol levels) were analysed on fresh meat (*n* = 6 LD/diet; *n* = 6 HL/diet).

### 2.3. Reagents

Sulfuric acid, hydrochloric acid, formic acid, perchloric acid, sodium nitrate, sodium hydroxide, potassium hydroxide, potassium chloride, sodium sulfate anhydrous, potassium dihydrogen phosphate, chloramine T trihydrate, trichloroacetic acid, 2-thiobarbituric acid, ethylenediaminetetraacetic acid (EDTA), propylgallate, 4-(dimethylamino)benzaldehyde, petroleum ether (b.p. 40–60 °C), methanol, ethanol, hexane, acetonitrile, 2-propanol, chloroform, sodium methoxide solution, amino acid standard, L-tryptophan, hydroxyproline, L-2-aminobutyric acid, and 1,1,3,3-tetraehoxypropane were purchased from Sigma-Aldrich (Sigma-Aldrich Co., St. Louis, MO, USA).

Supelco 37 comp. FAME mix, linoleic acid methyl ester isomer mix were purchased from Supelco (Supelco Analytical, Bellefonte, PA, USA), trans FAME mix K110 from Grace (Grace, Deerfield, IL, USA).

AccQ Fluor reagent kit and AccQ Tag eluent A concentrate was purchased from Waters (Waters Corp., Miliford, MA, USA). Boric acid was purchased from AFT (Bratislava, Slovakia), Kjeltabs from Velf Scientifica (Velf Scientifica srl, Usmate, Italy).

### 2.4. Chemical Assay of Muscles

Total protein content was determined by the Kjeldahl method [29]. Intramuscular fat content was determined by the Soxleth extraction method [30] by extraction with petrol ether (b.p. 40–60 °C). The ash content was determined by incinerating the samples in a furnace at 550 °C [30]. The dry matter content of different rabbit muscles was determined by drying samples at 60 °C, after which they were equilibrated to room humidity overnight, milled and passed through a 1-mm sieve, and further dried at 105 °C to a constant weight.

### 2.5. Amino Acid Profile

Hydrolysis of rabbit muscle samples for amino acid analysis was proceeded as described in Commission regulation (EC) No 152/2009. The amino acid assay was performed by AccQ Tag technology (Waters Corp., Miliford, MA, USA). For amino acid analyses in samples, a Shimadzu low pressure gradient high-performance liquid chromatography (HPLC) system (Shimadzu Corp., Kyoto, Japan) consisting of a solvent delivery module LC-10ATVP, auto injector SIL-10ADVP, column oven CTO-10ACVP, spectrofluorometric detector RF-10AXL, system controller SCL-10AVP, and online degasser DGU-14A was used. Workstation LC Solution (Shimadzu Corp., Kyoto, Japan) for HPLC system control and data collection was used. Amino acid derivatives were separated on Nova-Pak C18, 4 μm, 150 *×* 3.9 mm chromatography column (Waters Corp., Miliford, MA, USA) with a temperature of 37 °C. A total of 10 μL of derivatives was injected to separation. Separated derivatives were detected at E_x_ 250 nm–E_m_ 395 nm wavelengths. A gradient flow was used for separation of amino acids derivatives. Flow rate was set at 1 mL/min. Amino acids were identified by the retention times as compared to the retention times of the amino acid standard solution. The results were calculated by measuring the peak areas of the sample and standard solution for each amino acid.

The content of hydroxyproline in rabbit muscles samples was measured spectrophotometrically at 560 nm, as described by Stegemann and Stalder [31], and the content of tryptophan at 610 nm according to the procedure described by Miller [32]. Based on the obtained values, the tryptophan and hydroxyproline (T/H) ratio was calculated.

### 2.6. Fatty Acid Profile

Extraction of lipids for fatty acid analysis was performed with chloroform/methanol (2:1 *v/v*), as described by Folch et al. [33]. Fatty acid methyl esters (FAME) of the total lipids were prepared according to the procedure described by Christopherson and Glass [34].

The FAMEs were analysed using a gas chromatograph Shimadzu GC—2010 Plus (Shimadzu Corp., Kyoto, Japan) fitted with flame ionization detector. The separation of methyl esters of fatty acids was carried out on a capillary column Rt-2560 (100 m; 0.25 mm ID; 0.25 μm df) (Restek, Bellefonte, PA, USA) by temperature programming from 160 °C to 230 °C. The temperature of the injector was 240 °C, and the detector was 260 °C. The rate of flow of carrier gas (nitrogen)—0.79 mL/min. The injection volume was 1.0 μL.

The FAMEs were identified by comparing their retention times with those of the authentic standard mixtures: Supelco 37 comp. FAME mix; trans FAME mix K110; Linoleic acid methyl ester isomer mix. The relative content of each fatty acid in the sample was expressed as relative percentage of the sum of the fatty acids.

The average amount of each fatty acid was used to calculate the total of saturated (SFA), monounsaturated (MUFA), and polyunsaturated (PUFA) fatty acids. Lipid quality indices, atherogenic index (AI), and thrombogenicity index (TI), were calculated according to Ulbricht and Southgate [35]:

AI=[C12:0+(4×C14:0)+ C16:0]∕[ΣPUFA n6+ΣPUFA n3+ΣMUFA]

TI=[C14:0+C16:0+C18:0]∕[(0.5×ΣMUFA)+(0.5×ΣPUFA n6)+(3×ΣPUFA n3)+(ΣPUFAn3/ΣPUFAn6)]

The hypocholesterolemic/hypercholesterolemic (h/H) ratio was calculated according to Fernández et al. [36]:

h/H=(C18:1 n9+C18:2 n6+C20:4 n6+C18:3 n3+C20:5 n3+C22:5 n3+C22:6 n3)∕(C14:0+C16:0)

### 2.7. Cholesterol and Lipid Oxidation Levels

The cholesterol content in rabbit muscles was determined according to the method described by Polak et al. [37]. A high-pressure gradient HPLC system Varian ProStar (Varian, Inc., Palo Alto, CA, USA) was used for the cholesterol content determination. Cholesterol separation was performed on a LiChrospher 100 RP-18e (150 × 4.6 mm, 5 μm) chromatography column (Alltech Associates Inc., Deerfield, IL, USA), and the chromatogram was processed at a wavelength of 210 nm. As the mobile phase, a mixture of acetonitrile and 2-propanol (55:45) at a flow rate of 1.0 mL min−1 was used. The chromatography data were summarized and averaged from 6 replicate rabbit muscle samples for each treatment.

An assay of lipid oxidation (malondialdehyde (MDA) content) levels in the muscle samples was tested at two intervals: 24 h post-mortem and after 3 months following the slaughter; the determinations were carried out by the high-performance liquid chromatography method described by Mendes [38]. For this purpose, a high-pressure gradient HPLC system Varian ProStar (Varian, Inc., Palo Alto, CA, USA) with a ProStar 363 fluorescence detector was used. The separation of malondialdehyde–2–thiobarbituric acid (MDA-TBA) was performed on a Gemini C18 (250 × 4.6 mm, 5 μm) chromatographic column (Phenomenex, Inc., Torrance, CA, USA). The mobile phase consisting of 50 mM KH2PO4, methanol, and acetonitrile at a ratio of 72:17:11 was supplied at a flow rate of 1.0 mL min_−1_. The MDA-TBA compound was identified and quantified by measuring the fluorescence at E_x_ 525 nm–E_m_ 560 nm wavelengths. MDA-TBA compound was quantified by comparison between the peak area of the MDA-TBA compound in a sample and the peak area of this compound in standard solution.

### 2.8. Statistical Analysis

The study used samples from 3 groups of rabbits, each with 6 rabbit duplicates (*n* = 6 duplicate rabbits/diet); from one rabbit, two kinds of muscles were taken as samples for further analysis (*n* = 6 LD/diet; *n* = 6 HL/diet). Data analysis was performed by SPSS for Windows, version 25.0 (IBM Corp., Released 2017, Armonk, NY, USA). A one-way analysis of variance (ANOVA) test post-hoc (Fisher’s least significant difference test) was conducted to detect differences among treatments. A calculated *p* value of less than 0.05 (*p* < 0.05) was considered statistically significant.

## 3. Results

### 3.1. Chemical Composition of Rabbit Muscles

After the inclusion of freshwater *C. glomerata* biomass in the rabbits’ diet, the chemical composition of the different rabbit muscles was analysed (Table 2). According to recent findings, CG4 treatment resulted in the highest protein levels in LD muscles, reaching 22.17%. Compared to 8% biomass inclusion (CG8), protein levels in LD muscles from CG4 rabbits were higher by 1.81% (*p* < 0.05). However, after HL muscle analysis, the highest protein levels were discovered under SCD treatment. Therefore, the results were not significant (*p* > 0.05). The fat content of both muscles decreased as the biomass dosage in the diet increased. Therefore, the highest amount of fat was found in SCD, with a fat content of 1.62% in LD and 2.73% in HL, respectively. Compared to *C. glomerata* experimental diets, CG4 and CG8 fat contents in LD were respectively lower by 0.47% and 1.08%, and in HL, respectively, lower by 1.29% and 1.90%, compared to SCD (*p* < 0.05).

The highest ash content was determined in SCD-LD muscles, which was 0.07% greater than in CG8 (*p* < 0.05). In both LD and HL muscles, the dry matter (DM) levels differed significantly across all diets (*p* < 0.05). The highest DM was found in SCD, followed by CG4, and finally CG8. Both LD and HL muscles showed the same pattern of decreasing dry matter content with increasing dosages of *C. glomerata* (*p* < 0.05).

### 3.2. Amino Acid Profile of Rabbit Muscles

The profile of rabbit muscle amino acids is shown in Table 3. The predominant amino acid in both tested muscles from the identified 16 amino acids was glutamic acid. Its highest amount was determined in the muscles of CG4 rabbits, at 32.63 g/kg in LD and 29.61 g/kg in HL, respectively (*p* < 0.05).

The following essential amino acids were also identified: threonine, valine, methionine, isoleucine, leucine, phenylalanine, histidine, and lysine. The highest levels of threonine, valine, methionine, and isoleucine were discovered in LD muscles under 4% inclusion of *C. glomerata* biomass (CG4). In CG4-LD muscles, threonine levels were higher by 0.41 g/kg than in CG8, valine levels were higher by 0.86 g/kg than in CG8, methionine levels were higher by 2.02 g/kg, and 1.95 g/kg than in SCD and CG8, respectively, and isoleucine levels were higher by 0.84 g/kg than in CG8 (*p* < 0.05). The highest levels of leucine and phenylalanine were found in SCD-LD muscles; when compared to CG8 levels, they were 1.20 g/kg and 0.58 g/kg higher, respectively (*p* < 0.05). However, the opposite trend was discovered after HL sample analysis: higher leucine and phenylalanine levels were observed in the CG8 diet by 0.80 g/kg and 0.40 g/kg compared to SCD, respectively (*p* < 0.05). The highest concentration of histidine was found in SCD LD muscles (8.78 g/kg), and the lowest concentration was found in the mentioned muscles from CG8 (8.11 g/kg) (*p* < 0.05). The distribution of lysine concentrations in LD muscles according to the diets of rabbits was as follows: CG4 (16.71 g/kg) < SCD (15.86 g/kg) < CG8 (15.60 g/kg) (*p* < 0.05).

After studying both rabbit muscles, the amount of the remaining identified aspartic and tyrosine amino acids was found to be highest under CG4 treatment: respectively 18.43 g/kg in LD and 16.73 g/kg in HL; 6.69 g/kg in LD and 6.65 g/kg in HL (*p* < 0.05). Glycine levels in HL muscles were higher by 1.37 g/kg in CG8 compared to SCD (*p* < 0.05). Compared to SCD and CG4 diets, lower alanine contents were discovered in CG8 LD muscles by 0.98 g/kg and 1.02 g/kg, respectively (*p* < 0.05). However, after HL muscle analysis, alanine levels in SCD treatment were lower (8.87 g/kg) than in the CG4 (9.89 g/kg) and CG8 (10.14 g/kg) experimental diets (*p* < 0.05). The SCD rabbits’ LD muscles had the greatest amounts of arginine, which were 1.23 g/kg higher than those of the CG8 (*p* < 0.05). When comparing serine and proline in both analysed rabbit muscles, no significant differences between diets were found (*p* > 0.05).

The total amount of all amino acids in grammes per kilogramme of each muscle group was calculated (Table 3). The highest total amino acid amount in LD muscles was observed in the CG4 treatment (192.16 g/kg) and the lowest in the CG8 treatment (179.74 g/kg) (*p* < 0.05). Conversely, after HL muscle analysis, results showed that the highest total amino acid content was observed under CG8 treatment (176.13 g/kg), a comparably similar amount in CG4 (174.33 g/kg; *p* > 0.05), and the lowest amount in SCD (165.32 g/kg; *p* < 0.05). When comparing the total amount of amino acids between different muscles, a higher amount of it was found in LD muscles, assuming these muscles are also characterised by a higher protein content.

Tryptophan, a marker for muscle tissue, and hydroxyproline, a marker for connective tissue, were discovered in the muscles of rabbits (Table 4). The highest tryptophan levels were discovered in rabbits’ LD muscles under the CG4 diet (30.84 g/kg), followed by the SCD diet (29.40 g/kg), and the CG8 diet (25.28 g/kg) (*p* < 0.05). Tryptophan levels in HL muscles, in contrast, were higher in SCD by 3.48 g/kg and 2.25 g/kg compared to CG4 and CG8, respectively (*p* < 0.05). Hydroxyproline levels in LD muscles ranged from 6.30 g/kg in CG4 to 7.71 g/kg in SCD and 8.85 g/kg in CG8 (*p* < 0.05). Significant differences in hydroxyproline levels in HL muscles were found only between SCD and CG4 treatments, with its levels being 2.98 g/kg higher in SCD compared to CG4 (*p* < 0.05). The CG4 treatments’ LD muscles exhibit the greatest tryptophan/hydroxyproline ratio (T/H). Compared to other diets, the LD muscle ratio of CG4 (4.91) was 1.3 times higher than in SCD and even 1.7 times higher than in CG8 (*p* < 0.05). When comparing T/H in HL rabbit muscles, no significant differences between diets were observed (*p* > 0.05).

### 3.3. Lipids in Rabbit Muscles

#### 3.3.1. Fatty Acid Profile

The fatty acid profiles of rabbit muscles under the influence of SCD, CG4, and CG8 diets are presented in Table 5. Overall, the most saturated fatty acids (SFA) were found in the SCD muscles (34.38–35.61%), while the CG4 and CG8 muscles were dominated by polyunsaturated fatty acids (PUFA) (34.18–38.92%). Comparing the total amount of SFA in LD muscles, SCD had 1.76% more SFA compared to CG8, and in HL muscles, the SFA amount was lower by 1.23% and 1.97%, respectively, in CG8 compared to SCD and CG4 (*p* < 0.05). The dominant individual SFA in all muscles was palmitic acid (C16:0); the highest amounts of this acid were observed in SCD-LD and HL muscles, at 26.81% and 25.21%, respectively. Higher concentrations of capric acid (C10:0) in LD muscles, lauric acid (C12:0) in LD and HL muscles, myristic acid (C14:0) in HL muscles, and pentadecylic acid (C15:0) in LD and HL muscles were found in SCD compared to CG8 (*p* < 0.05). Margaric acid (C17:0) was mostly found in SCD LD and HL muscles, with its levels being 0.06% equally higher in LD and 0.03% and 0.05% greater in HL compared to CG4 and CG8, respectively (*p* < 0.05). Stearic acid (C18:0) dominated in CG8 LD and HL muscles (7.57% and 8.25%, respectively). Greater amounts of arachidic acid (C20:0) were found in muscles under CG diets: 0.10% in the LD muscles of CG8 and 0.09% in the HL muscles of CG4. Behenic acid (C22:0) was dominant in CG8 HL muscles. Its concentration, compared to SCD and CG4 diets, was 0.09% and 0.08% higher in CG8-HL muscles, respectively (*p* < 0.05).

The total amount of monounsaturated fatty acids (MUFA) revealed that the SCD diet’s LD and HL muscles had most of these FAs (31.18% and 31.12%, respectively). Compared to other treatments, the MUFA content in SCD was higher by 4.14% and 6.88% in LD and by 5.22% and 8.07% in HL, compared to CG4 and CG8, respectively (*p* < 0.05). Myristoleic acid (C14:1 n7), hexadecanoic acid (C16:1 n9), and palmitoleic acid (C16:1 n7) concentrations were found to be higher in SCD LD and HL muscles compared to CG8 (*p* < 0.05). Margaric acid (C17:1 n9) was higher in SCD muscles as well, compared to both *C. glomerata*-based diets (CG4 and CG8) (*p* < 0.05). A lower amount of elaidic acid (C18:1 n9t) was discovered in SCD HL muscles; it was lower by 0.07% and 0.09% compared to CG4 and CG8, respectively (*p* < 0.05). According to the different diets, oleic acid (C18:1 n9) concentrations in both LD and HL rabbit muscles were distributed like this: SCD < CG4 < CG8. Under the influence of CG8, the same concentration (1.16%) of vaccenic acid (C18:1 n7) was found in the LD and HL muscles of rabbits. When *C. glomerata* inclusion increased, eicosenoic acid (C20:1 n9) decreased. However, only one significant difference was found between the mentioned acid concentration in SCD and CG8 HL muscles: it was higher by 0.04% in SCD compared to CG8 (*p* < 0.05).

Total PUFA content in muscle increased with increasing dosages of *C. glomerata* in the rabbit diet. PUFA content reached 35.99% and 38.92% in CG8 LD and HL muscles, respectively; 34.18% and 36.08% in CG4 muscles; and only 30.81% and 32.54% in SCD muscles (*p* < 0.05). However, a lower concentration of linolelaidic acid (C18:2 n6t) was obtained in CG8 HL muscles (0.06%), while in CG4 HL muscles it was higher by 0.02% (0.08%) (*p* < 0.05). The greatest content of α-linolenic (C18:3 n3) acid was discovered in both LD and HL muscles on the SCD diet. This fatty acid was observed in 2.90% and 3.01% of the samples, respectively. In comparison to CG4 and CG8, the α-linolenic acid content was found to be nearly 1.6 times lower in the LD muscles of CG8 and about 1.2 and 1.8 times lower in the HL muscles of CG4 and CG8, respectively (*p* < 0.05). Eicosatrienoic acid (C20:3 n3) was not defined at all in LD muscles under CG8 treatment. However, after HL muscle analysis, the concentration of the mentioned acid was the highest in SCD compared to diets supplemented with *C. glomerata* (*p* < 0.05). In contrast, remaining significant differences were found when individual PUFAs (eicosadienoic (C20:2 n6), dimoho-γ-linolenic (C20:3 n6), arachidonic (C20:4 n6), eicosapentaenoic (C20:5 n3), adrenic (C22:4 n6), and docosapentaenoic (C22:5 n3) acids) were higher in CG8 muscles compared to other diets (*p* < 0.05). Eicosadienoic acid levels were 0.04% and 0.05% greater in CG8 LD muscles than in SCD and CG4 muscles, as well as 0.05% higher than in CG4 HL muscles (*p* < 0.05). The dimoho-γ-linolenic acid concentrations in both LD and HL muscles were distributed descendingly, with the lowest concentrations in SCD muscles, higher in CG4, and the highest in CG8 (*p* < 0.05). Arachidonic acid was several times higher in CG8 muscles compared to SCD and CG4 diets: 3.3 and 2.1 times higher in LD muscles, respectively, and 2.7 and 1.9 times higher in HL muscles, respectively (*p* < 0.05). The lowest levels of eicosapentaenoic acid were determined in SCD muscles, which reached only 0.06% and 0.05% in LD and HL, while in CG4 it was 0.11% and 0.08%, and in CG8 even 0.14% and 0.12% (*p* < 0.05). Although adrenic acid was mainly detected in the muscles of CG8, the next highest level of this acid was discovered in SCD, followed by CG4 (*p* < 0.05). A greatly higher concentration of docosapentaenoic acid was found in CG8 muscles compared to SCD and CG4: 3.4 and 1.9 times higher of the mentioned fatty acid was discovered in LD muscles, and 2.6 and 1.6 times more in HL muscles, respectively (*p* < 0.05).

When evaluating the entire fatty acid profile, the most unidentified fatty acids were found in CG8 muscles. Such unidentified fatty acid levels in LD and HL muscles of CG8 reached 5.96% and 4.96% from all fatty acids, while lower levels were obtained in CG4 muscles (3.70% and 3.02%) and in SCD (2.48% and 2.11%) (*p* < 0.05).

The PUFA/SFA ratio was calculated (Table 5). This ratio proportionally increased when the *C. glomerata* biomass in the rabbit diet increased. The distribution in the LD and HL muscles was as follows: CG8 (1.06% and 1.17%) < CG4 (0.97% and 1.03%) < SCD (0.87% and 0.95%) (*p* < 0.05).

The addition of *C. glomerata* biomass to the diet had no influence on the level of omega-3 in the muscles of rabbits (*p* > 0.05). The omega-3/omega-6 ratio was not affected by CG diets as well (*p* > 0.05). However, the inclusion of biomass in the rabbit diet affected the omega-6 concentration in muscles, which was found to be 3.25% and 4.79% higher in CG4 and CG8 LD muscles and 3.72% and 6.45% higher in HL muscles compared to SCD, respectively (*p* < 0.05).

Atherogenic (AI) and thrombogenicity (TI) indexes, as well as the hypocholesterolemic/hypercholesterolemic ratio (h/H), were calculated. The AI index in LD muscles was not affected by CG experimental diets (*p* > 0.05). However, differences were obtained after evaluating the HL muscles, where a 0.08 lower index was obtained in CG8 compared to the same values obtained in SCD and CG4 (0.54) (*p* < 0.05). Analogous values of TI index were observed in SCD and CG4 LD muscles (0.86), so the means did not differ significantly between diets (*p* > 0.05). Therefore, a higher TI index was found in CG4 HL muscles (0.85), which was greater compared to the analogue values (0.80) of SCD and CG8 (*p* < 0.05). The highest h/H ratio was obtained in both LD and HL CG8 muscles. Compared to SCD, this ratio was found to be 0.26 higher in CG8 LD muscles, and compared to SCD and CG4, it was 0.30 and 0.28 higher in CG8 HL muscles, respectively (*p* < 0.05).

#### 3.3.2. Malondialdehyde (MDA) and Cholesterol Levels

**Figure 2:**
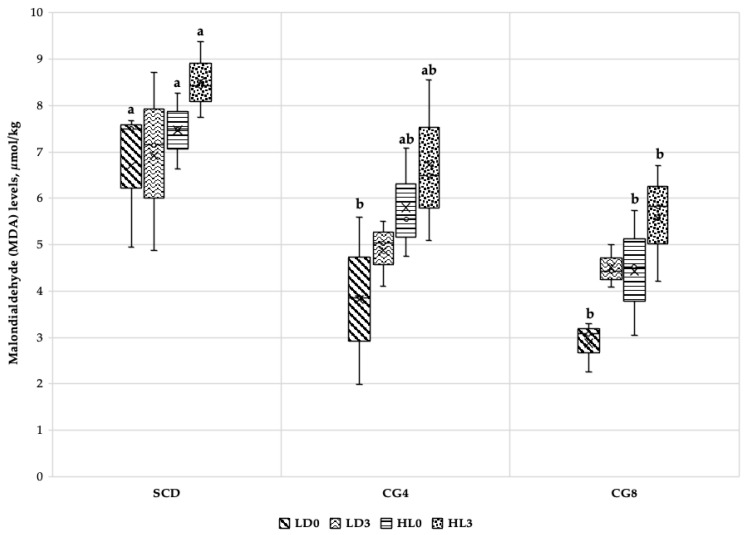
Impact of freshwater *C. glomerata* biomass inclusion in rabbit diets on malondialdehyde (MDA) levels (μmol/kg) in fresh (LD0 and HL0) and stored for 3 months (LD3 and HL3) rabbit muscles. SCD, standard compound diet; CG4, standard compound diet + 4% *C. glomerata* biomass; CG8, standard compound diet + 8% *C. glomerata* biomass. Columns with the same pattern but different superscript letters (a–b) differ significantly (*p* < 0.05).

Malondialdehyde (MDA) levels were examined in rabbit fresh muscles 24 h after slaughter and in rabbit muscles stored for 3 months at −18 °C in a freezer (Figure 2). The results were distributed proportionally: as the dose of *C. glomerata* increased, the level of lipid oxidation decreased. A lower level of MDA was obtained first when examining the LD fresh muscles (LD0) of CG8, then CG4. Compared with SCD, MDA levels in LD0 muscles of CG8 and CG4 were 3.83 μmol/kg and 2.90 μmol/kg lower, respectively (*p* < 0.05). However, *C. glomerata* biomass inclusion did not have any significant impact on lipid oxidation levels in stored LD (LD3) muscles (*p* > 0.05). The same trend was observed when MDA levels were higher in SCD diets and lower in CG diets after fresh (HL0) and stored (HL3) HL muscle evaluation. MDA levels were respectively higher by 3.04 μmol/kg and 2.94 μmol/kg in SCD HL0 and HL3 muscles compared to CG8 (*p* < 0.05).

**Figure 3:**
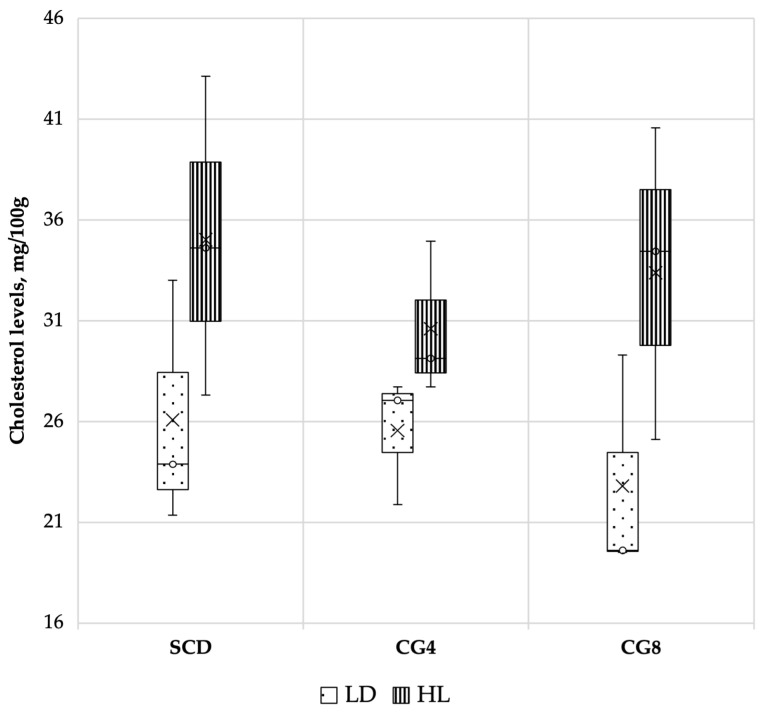
Impact of freshwater *C. glomerata* biomass inclusion in the diet of rabbits on cholesterol levels (mg/100 g) in different rabbit muscles (LD and HL). SCD, standard compound diet; CG4, standard compound diet + 4% *C. glomerata* biomass; CG8, standard compound diet + 8% *C. glomerata* biomass.

The impact of freshwater *C. glomerata* biomass inclusion in the diet of rabbits on cholesterol levels in different muscles is presented in Figure 3. Nevertheless, the CG diet had no significant impact on levels of cholesterol in the LD and HL rabbit muscles (*p* > 0.05).

## 4. Discussion

### 4.1. Dry Matter of Rabbit Muscles

Meat, in general, is exceedingly perishable due to its high moisture content, causing rapid quality deterioration and bacteria growth if not properly maintained [39,40]. However, *C. glomerata* inclusion in rabbits during our study significantly reduced the DM content of both tested rabbit muscles, thereby directly increasing muscle moisture. Hafsa et al. [41] obtained very comparable results to ours when they acquired rabbit meat with nearly 3% higher moisture in their test with the freshwater algae *C. aegagropila*, which belongs to *Cladophora* sp. Following ash determination in rabbit muscles, the same pattern was observed, with ash decreasing as *C. glomerata* inclusion in diet increased. Nevertheless, during LD muscle analysis, only significant results were observed. Given these indicators, it is to be assumed that meat has a high percentage of water, so it is critical to follow the instructions and properly store and handle meat-based foods to avoid deterioration. Thus, achieving functional food starts with the fundamentals. Food functional qualities, according to Fogliano and Vitaglione [42], can be included in a variety of ways: 1. by incorporating a functional component into a traditional food matrix to produce enriched food with a higher and unusual nutrient composition; 2. by manipulating food through technological processes, such as boosting the formation of compounds with specific biological activities or removing a potentially negative component; and 3. by enhancing functional nutrients or compounds through animal feeding, special growing conditions, or genetic manipulation. In our case, we use the third way by supplementing rabbits’ feed with freshwater biomass, which contains a variety of biologically active compounds.

### 4.2. Proteins and Amino Acid Profiles of Rabbit Muscles

Based on its nutritional and dietetic features, rabbit meat is recognised as an exceptional-quality protein in human diets [24]. It contains a significant amount of protein (approximately 22%) and an excellent essential amino acid profile [20]. Considering *Cladophora* species, it can be utilised as a source of protein in animal nutrition since its biomass has a protein level ranging from 10 to 25%, which is comparable to other feed materials [2]. In a recent study, we evaluated how different dosages of *C. glomerata* biomass in rabbit diets can affect protein content and amino acid composition in their muscles. The highest protein levels (22.17%) were observed in the LD muscles of rabbits that were receiving 4% *C. glomerata* treatment in their diets. However, after doubling the macroalgal biomass dosage to 8% in rabbit diets, the protein content in LD muscles was discovered to be the lowest compared to both the 4% dosage of biomass and the standard compound feed. Abu Hafsa et al. [41] examined freshwater macroalgae from natural water resources in Egypt for inclusion at 4% in rabbit diets. They used algae similar to our study’s *C. glomerata* freshwater macroalgae, *C. aegagropila*, and the results demonstrated that this type of inclusion resulted in 18.63% of protein in rabbit meat in general, which is slightly lower to our observations. Nevertheless, it is important to note that the protein content of *C. aegagropila* freshwater macroalgae from Egypt was only 10.44%, while *C. glomerata* collected from the Lithuanian river Šventoji that we used in our study contained 22.36% of protein [27]. According to our findings, freshwater macroalgae like *C. glomerata* can be utilised as a protein source in rabbit diets. Since rabbit meat is an excellent source of animal-derived protein that can partially meet human daily amino acid requirements [43], *C. glomerata* biomass in general can improve the functionality of meat even further. Therefore, given the global challenges we confront with livestock today, including the expected shortage of traditional protein sources in animal feed production, freshwater macroalgae could be one of the solutions for more sustainable, alternative, and functional feed materials [16].

Animal husbandry produces a large amount of sustainable protein for human consumption [44]. For example, rabbit meat is high in essential amino acids in addition to being high in protein [24]. Although animal production quality is directly related to the animal’s diet, it is crucial to highlight that amino acids used in various types of feed materials can increase animals’ nutrient digestibility, compensate for nutrient deficiencies, and improve feed quality and the final composition of animal production [4]. That implies that a healthy animal produces a high-quality product. Amino acids serve as protein building blocks, as an energy source, and as precursors for biologically active molecules [43]. Whether an amino acid is labelled as essential or non-essential, animals and humans require sufficient levels of all amino acids to meet their metabolic demands. Animals, for example, cannot synthesise essential amino acids and must consequently receive them from their diet [45]. Few scientists, including our prior study, examined the amino acid profile of *Cladophora* biomass, and the findings confirmed a highly remarkable new raw material that might potentially be utilized in animal feed [13,16]. Taking the rabbit’s meat amino acid profile composition into account, it contains more lysine, threonine, valine, isoleucine, leucine, and phenylalanine compared to other meats [46]. The same essential amino acids—threonine, valine, methionine, isoleucine, leucine, phenylalanine, histidine, and lysine—were identified in rabbit muscles during a recent study. In most cases, the diet supplemented with 4% *C. glomerata* biomass had the greatest impact on the levels of the aforementioned amino acids. After this treatment, rabbit LD muscles had the highest levels of threonine, valine, methionine, lysine, and isoleucine. In this way, rabbit LD muscle proteins have a high biological value due to their enhanced and balanced essential amino acid content and their simplicity of digestion, which was affected by *C. glomerata* biomass in the rabbit diet. Methionine, for example, is one of the most limiting amino acids and is essential for protein synthesis in the body, preserving its advantageous function as a methyl group donor [47,48]. The daily dietary intake of methionine is similar to that of other essential amino acids [49]; however, it can also be affected by human physiological phases such as pregnancy or infancy rather than only the availability of methyl donors or acceptors and cysteine [50]. The pattern of another essential amino acid lysine levels in rabbit LD muscles according to diet was as follows: CG4 < SCD < CG8. Lysine, along with leucine, is significant since it generates ketone bodies, which serve as an alternate energy source in our bodies [51]. However, *C. glomerata* inclusions did not increase leucine-phenylalanine levels in LD muscles. Conversely, higher levels of the mentioned essential amino acids were found in the HL muscles of rabbits under 8% macroalgal biomass treatment. Histidine was slightly higher in LD muscles under standard compound diet treatment compared to 8% *C. glomerata* inclusion. However, in general, the nutritional value of meat proteins was demonstrated in most ways to be higher in rabbits treated with 4% *C. glomerata* inclusion, based on a higher proportion of essential amino acids in LD muscles. Considering essential amino acid concentrations are frequently used to estimate the biological value of proteins, the ability to meet customer demand for this kind of acid is crucial [52].

In recent research, conditionally essential glutamic acid was the most abundant amino acid in LD and HL muscles across all treatments. This amino acid is mostly prevalent in rabbit meat. Morshdy et al. [43] investigated the LD muscles of New Zealand and Californian breed rabbits (the same breed as in our study) and supported our results by discovering that the amino acid profile was dominated by the same glutamic acid. The remaining conditionally essential amino acids are unevenly distributed: higher levels of glycine in HL muscles were obtained when rabbits were supplemented with an 8% macroalgae biomass dose compared to standard feed; on the contrary, a higher amount of arginine was found in SCD LD muscles compared to CG8. *C. glomerata*, which had no effect on the levels of serine and proline in different rabbit muscles, according to our findings. Nonessential amino acids were distributed as follows: lower alanine levels were discovered in CG8 LD muscles compared to the remaining diets; aspartic acid levels were the highest in both analysed muscles under CG4 treatment.

The current study’s findings, in general, point to the dietetic properties of rabbit meat, not only due to its high fraction of essential amino acid content but also due to its increased total amino acid content. A total of 4% inclusion of *C. glomerata* increased the total amino acid content in LD muscles (192.16 g/kg); a doubled dosage of 8% biomass in rabbit diets increased the same indicator in HL muscles (176.13 g/kg). First, amino acid compositions in meat are determined by diverse amino acid syntheses, which are associated with distinct biological stages of animals [53]. However, the feed can play a key role as well. For example, in *C. glomerata* biomass collected from Lithuanian rivers, the total amino acid content can vary from 103.36 to 140.99 g/kg [16]. Thus, only the essential amino acid content can vary from 41.60 to 55.40 g/kg, which is about 40% of the total amount of amino acids determined according to our previous study. Regardless of the ultimate result, the taste of the food will remain one of the primary factors shaping the consumer’s daily choices and habits [54]. As a result, it is important to recognize that amino acids can lead to specific tastes in food, including meat [55]. So, the advantageous amino acid profile of *C. glomerata* macroalgal biomass could affect the taste of rabbit meat. Threonine, serine, proline, glycine, and alanine in biomass may activate a sweet flavour; valine, isoleucine, leucine, phenylalanine, histidine, lysine, and arginine may activate a bitter flavour; phenylalanine, tyrosine, and alanine may activate a sour flavour; and glutamic and aspartic acid may activate an umami flavour [56].

Meat quality is a complex notion; for example, one of the most crucial elements for consumers is meat tenderness. One of the variables that directly affect and decrease meat tenderness is connective tissue proteins [57]. The hydroxyproline content of muscles is commonly used as a marker of connective tissue, whereas tryptophan content is used as a marker of muscle tissue. Tryptophan levels in LD muscles were enhanced by the 4% inclusion of *C. glomerata* macroalgal biomass in rabbit diets. When the dosage of *C. glomerata* in the diet was increased twofold (8%), tryptophan levels were marginally decreased and were lower compared to the standard compound diet. However, after HL muscle analysis, the distribution of results differed from that of LD muscles, with tryptophan being the most abundant in SCD (26.27 g/kg), followed by CG8 (24.02 g/kg), and finally CG4 (22.79 g/kg). Another identified amino acid, hydroxyproline, is present primarily in connective and bone tissue and contributes up to 10% of collagen molecules [57]. So, hydroxyproline is considered to be an excellent marker for evaluating meat quality [58]. The lowest hydroxyproline levels were observed in both the LD and HL muscles of rabbits fed a diet containing 4% *C. glomerata* biomass. It is significant since rabbit muscles from the CG4 diet had higher tryptophan and lower hydroxyproline levels, indicating that including 4% *C. glomerata* in rabbit diets can improve meat quality. To be more specific, the tryptophan-to-hydroxyproline ratio (T/H) is by far the most important criterion for meat quality and a protein quality indicator [58,59]. The higher this indicator, the greater the meats nutritional value, as well as the overall amount of muscle tissue and proteins, as well as essential amino acids. In our case, the CG4 diet enhanced the T/H ratio in LD muscles (4.91); thus, 8% biomass inclusion, on the contrary, decreased the T/H ratio and was the lowest (2.86) when compared to other treatments. *C. glomerata* inclusion by 4% in rabbit diets can increase the T/H ratio, which indicates the potential to enhance rabbit meat with a higher biological value. Therefore, according to the obtained results, any *C. glomerata* inclusion did not impact the T/H ratio in HL rabbit muscles.

### 4.3. Lipids and Fatty Acid Profile of Rabbit Muscles

Meat and meat-based products are rarely mentioned as unfavourable due to their high fat and calorie content, as well as higher SFA and cholesterol levels, which are usually linked to cardiovascular disease, obesity, and diabetes [24]. A number of these detrimental nutrients can be reduced by carefully selecting the meat parts eaten, manipulating productive factors, primarily feeding, and manipulating the carcass post-mortem. Furthermore, rabbit meat is highly regarded globally for its excellent nutritional features, with lower fat, fewer saturated fats, and lower cholesterol levels compared to other commonly consumed meats [24]. To be precise, rabbit meat is lower in fat (9.2 g/100 g) and cholesterol (56.4 mg/100 g) compared to chicken, beef, and pork [60]. Due to its low-fat content, rabbit meat has a lower energy value than red meats. According to other researchers, fat content can range from 0.6 to 14.4% depending on the carcass portion (average value of 6.8%), with the loin being the leanest part of the rabbit’s carcass (1.2% lipids) [61]. In our case, synergism between alternative freshwater *C. glomerata* inclusion in rabbit feed and rabbit muscles, which are already considered functional foods, lowered the fat content in both LD and HL muscles. It was revealed that the greater the dosage of *C. glomerata* in the diet, the lower the fat accumulation in the rabbit’s muscles. Fat distribution in rabbits’ muscles: LD muscles SCD (1.62%) < CG4 (1.15%) < CG8 (0.54%); HL muscles SCD (2.73%) < CG4 (1.44%) < CG8 (0.83%). In aquaculture, more feeding studies with *C. glomerata* have been performed compared with other animal species. When we compared our findings to those of Promya and Chitmanat [19], who supplemented African sharptooth catfish (*Clarias gariepinus*) diet with a 5% dosage of *C. glomerata* biomass produced under artificial conditions, they discovered that the final fish muscles had less fat than the standard diet-fed ones. This demonstrates that not only our findings but also those of other researchers support the direct decrease of fat in muscles by supplementing feed with *C. glomerata* biomass. This is particularly crucial for individuals who prefer leaner meat in their diets. These preferences are mostly linked to the extraordinary leanness of the meat, the healthiness of the lipid profile, the micronutrient balance attributes, and the low level of cholesterol [62].

Rabbit meat has an excellent lipid profile since it is low in cholesterol and SFAs while being high in PUFAs, including a balanced ratio of essential omega fatty acids [63]. However, considering the higher PUFA content, the meat becomes more sensitive to oxidative degradation, which can directly affect not only the shelf life of the product but also its final sensory properties [20]. As a result, the degree of lipid oxidation in the final product is substantial, and to assure the stability of lipids in meat, we can first enhance animal feed with antioxidant-rich raw materials [63]. *C. glomerata* has been identified as having antioxidant properties due to the presence of specific phenols, pigments, and antioxidant activities [15]. It is reflected in our study, where we supplemented this type of biomass into rabbit diets and found a decrease in malondialdehyde (MDA) levels in fresh LD and HL muscles and stored HL muscles; the decrease was proportionally higher with higher biomass inclusion. However, *C. glomerata* biomass inclusion did not affect MDA levels in stored (3-month) LD muscles. Overall, the findings suggest that including macroalgae in rabbit diets might improve the quality of the meat and enhance its stability.

The link between health and diet is becoming increasingly crucial in shaping consumer behaviours. Nonetheless, meat is commonly linked with cholesterol levels in it, and while it is now accepted that dietary cholesterol consumption has a minor impact on plasma cholesterol, this is another unfavourable factor in meat’s nutritional perception for consumers [64]. Rabbit meat contains the least cholesterol of any common meat (47.0 mg/100 g in LD muscles and 61.2 mg/100 g in HL muscles) [24]. In our case, cholesterol levels in LD muscles varied from 22.81 to 26.08 mg/100 g and in HL from 30.60 to 35.02 mg/100 g. Up to a certain level, diet can influence the accumulation of cholesterol in rabbit tissues such as muscle. Nonetheless, since no significant differences between treatments were observed, the *C. glomerata* supplemented diet had no significant impact on cholesterol levels in the LD and HL rabbit muscles.

There is expanding scientific evidence that supports the concept that certain foods and dietary components provide physiological and psychological benefits along with basic nutrients [65,66,67,68]. In addition to these foods, new ones are being developed to improve or incorporate these health-promoting elements. Fatty acids (FAs), for example, are advantageous nutrients because their composition has a significant influence on a balanced, healthier diet because individual FA alter plasma lipids in different ways. The FA composition of rabbit meat is characterized by its high PUFA concentration; however, the FA profile may also be altered via diet. When it comes to algae, FAs are regarded as one of its most significant and biologically active components, particularly PUFAs, which are essential for human and animal health. The FA profile of different groups in the *C. glomerata* macroalgal biomass from natural sources used in our study was distributed as follows (% from the total FA content): saturated fatty acids (SFA) more than 50%, monounsaturated fatty acids (MUFA) 27.34–28.39%, and polyunsaturated fatty acids (PUFA) 6.48–11.71%. In the recent study, the accumulation of different FAs in rabbits’ muscles was distributed as follows: SFA in LD muscles: SCD (35.61%) < CG4 (35.21%) < CG8 (33.85%), in HL muscles CG4 (35.12%) < SCD (34.38%) < CG8 (33.15%); MUFA in LD muscles: SCD (31.18%) < CG4 (27.04%) < CG8 (24.30 %), and in HL muscles SCD (31.12%) < CG4 (25.90%) < CG8 (23.05 %); PUFA in LD muscles: CG8 (35.99%) < CG4 (34.18%) < SCD (30.81%), in HL muscles CG8 (38.92%) < CG4 (36.08%) < SCD (32.54%). Fike et al. [69] found a partially similar effect of macroalgae on the FA composition of meat when they fed lambs *Ascophyllum nodosum* (brown macroalgae) extract at 3.0 kg/ha or 1% DM and discovered a decrease in total SFA and a non-significant increase in unsaturated FAs. In our case, *C. glomerata* inclusions in rabbit diets (4% and 8%) gradually decreased SFA and MUFA levels in rabbit muscles while significantly enhancing PUFA levels.

Since humans are unable to synthesize essential PUFAs, they must receive them through food. Functional food elements include PUFAs, especially long-chain, highly unsaturated FAs with an omega-3 structure [9]. Given their role in metabolism, it’s not odd that they’ve been attributed to a variety of health advantages, including antibacterial, anti-inflammatory, antioxidant, cardiac disease prevention, and tumour growth suppression [70,71,72,73]. Looking deeper into the PUFA profile obtained when rabbits were fed different dosages of macroalgal biomass, the trend observed revealed that the higher the *C. glomerata* inclusion, the higher the PUFA deposition in rabbit muscle. The predominant fatty acid from all PUFAs was linoleic acid (C18:2 n6), but no significant impact was discovered when comparing its levels between diets. Individual omega-6 FAs (eicosadienoic (C20:2 n6), dimoho-γ-linolenic (C20:3 n6), arachidonic (C20:4 n6), and adrenic (C22:4 n6)), as well as some omega-3 FAs (eicosapentaenoic (C20:5 n3) and docosapentaenoic (C22:5 n3)), were significantly increased in both analysed rabbit muscles after treatment with 8% *C. glomerata* inclusion. It is commonly acknowledged that rabbits and other non-ruminants may directly convert dietary fatty acids into lipids in adipose and muscular tissues [74]. As a result, dietary lipid content has a substantial influence on fatty acid composition. Although the lipid concentration of *C. glomerata* macroalgae is lower compared to microalgae or oily land plants like rapeseed or flaxseed, such biomass is distinguished by a more nutritionally advantageous quality of lipids, as evidenced by our findings and final rabbit meat production. Furthermore, since modification of the rabbit diet is notably effective in increasing levels of PUFA, this kind of functional rabbit meat intake might become a valuable source of bioactive compounds for consumers. FAs, for example, have a crucial role in human metabolism, health, and diseases as biological compounds [75].

The PUFA/SFA ratio is a key metric that is frequently used to determine the effect of diet on cardiovascular health (CVH). The following theory was proposed based on this indicator: Low-density lipoprotein cholesterol (LDL-C) and serum cholesterol levels can be reduced by consuming PUFAs, whereas all SFAs lead to high serum cholesterol levels [75]. As a result, the greater this ratio, the greater the beneficial effect. In our trial, the results again reflected the following trend: the higher the dosage of *C. glomerata*, the higher the value of the indicator. So, this ratio increased proportionally when the *C. glomerata* biomass in the rabbit diet increased by being distributed in the LD and HL muscles, as follows: CG8 (1.06% and 1.17%) < CG4 (0.97% and 1.03%) < SCD (0.87% and 0.95%). Furthermore, our findings suggest that including *C. glomerata* in feed might enhance rabbit meat, which has a stronger influence on humans’ CVH when consumed.

Therefore, considering the final amount of omega-3 in the muscles, macroalgal biomass did not impact this indicator; however, since the 8% dosage directly affected most individual omega-6 FAs, a greater total amount of such FAs was obtained in the muscles of the CG8 diet. Overall, *C. glomerata* supplementation had no effect on the omega-3/omega-6 ratio.

Another metric evaluated in rabbit muscles was its atherogenicity index (AI), which reflects the correlation between total SFA and unsaturated FA contents. The primary classes of SFAs (lauric (C12:0), myristic (C14:0), palmitic (C16:0), and octadecanoic (C18:0)) are pro-atherogenic as they promote lipid adherence to cells of the circulatory and immune systems [76,77]. As a result, eating foods with lower amounts of AI can lower total cholesterol and LDL-C levels in human plasma [78]. The AI index in LD muscles was not affected by *C. glomerata* experimental diets, but on the contrary, 8% macroalgal biomass inclusion influenced HL muscles, which had the lowest AI levels compared to the remaining diets. As well as AI and PUFA/SFA ratio, another index, the thrombogenicity index (TI), should be lower to be beneficial to CVH. To be more explicit, TI defines the link between pro-thrombogenic FAs (C12:0, C14:0, and C16:0) and anti-thrombogenic FAs (MUFAs and omega 3, 6), which reflects the thrombogenic potential of FAs [35]. When comparing LD muscle TI values, nonetheless, *C. glomerata* had no significant impact on them. Only the inclusion of 4% biomass increased the TI value (0.85) in HL muscles; TI values in SCD and CG8 were analogues. Even though lower AI and TI values indicate greater nutritional quality and may reduce the risk of coronary heart disease (CVD), no organization has yet provided recommended values for these indicators. The hypocholesterolemic/hypercholesterolemic (h/H) ratio, which is more accurate than the PUFA/SFA ratio, also reflects FA effect on CVD. However, unlike AI and TI, foods with higher h/H indices are more nutritionally desirable [79]. According to our findings, the higher the *C. glomerata* inclusion, the higher the h/H obtained. The h/H ratio in LD muscles was 1.03 and 1.13 times higher in LD and 1.00 and 1.14 times higher in HL muscles with 4% and 8% macroalgal inclusion, respectively, as compared to SCD. Our findings suggest that supplementing the rabbit diet with *C. glomerata* biomass allows us to obtain rabbit meat, which can significantly reduce the risk of heart disease and is more nutritionally desirable.

## 5. Conclusions

In the production of enhanced functional rabbit meat, dietary supplementation with 4% and 8% inclusions of freshwater *C. glomerata* macroalgal biomass from natural resources might be considered not only beneficial but also a more sustainable nutritional strategy:

- A 4% inclusion can significantly increase protein and total amino acid levels in rabbits’ muscles while also increasing levels of essential amino acids (threonine, valine, methionine, lysine, and isoleucine) and tryptophan but decreasing hydroxyproline. As a result of their improved and balanced essential amino acid content, rabbit muscle proteins have a higher biological value, resulting in simpler digestion.
- Synergism between alternative freshwater *C. glomerata* inclusion in rabbit feed can lower the fat content of rabbits’ muscles; the greater the dosage of biomass in the diet, the lower the fat accumulation. Since biomass has a reduced fat content, it can reduce lipid oxidation levels in both fresh and stored muscles.
- Inclusions of 4% and 8% can gradually decrease SFA and MUFA levels in rabbit muscles while significantly enhancing PUFA, which indicates a more nutritionally advantageous quality of lipids. Rabbit meat under both *C. glomerata* treatments can result in increased heart disease prevention abilities, as observed PUFA/SFA and h/H values were greater, and TI and AI were lower.

## Figures and Tables

**Table 1:** Ingredients in rabbit feed and chemical composition of a standard compound diet and diets supplemented with different dosages of *C. glomerata* biomass (52–122 days old).

	Diet ^3^		
Ingredient (%) ^1,2^	SCD	CG4	CG8
Corn	3.00	3.13	3.44
Barley	18.00	18.00	18.00
Oats	25.00	25.00	25.00
Sunflower meal	13.22	11.97	9.97
Linseed meal	1.00	1.00	1.00
Soy meal	3.72	3.00	3.00
Vegetable oil	1.00	1.00	1.00
Beer east	2.00	2.00	2.00
Hay	29.26	27.10	24.79
*C. glomerata*	–	4.00	8.00
Antimycotoxin	0.30	0.30	0.30
Vitamin-mineral premix	3.50	3.50	3.50
Total	100	100	100
Chemical composition (%)			
DE (MJ/kg)	10.49	11.13	12.16
Crude protein	17.54	17.53	17.54
Crude fibre	13.56	14.39	15.05
Ash	10.03	10.37	10.31
Ether extract	3.12	3.20	3.10
NDF	32.49	34.19	35.89
ADF	19.71	20.12	20.73
ADL	4.89	4.94	5.15

Note: ^1^ Vitamin and mineral premix (per kg of feed): vitamin A 10.08 TV, vitamin D_3_ 1.14 TV, vitamin E 50.30 mg, vitamin K_3_ 0.99 mg, vitamin B_1_ 3.71 mg, vitamin B_2_ 2.80 mg, vitamin B_5_ 9.80 mg, vitamin B_12_ 0.01 mg, nicotinic acid 20.40 mg, folic acid 0.22 mg, choline chloride 170.00 mg, Mg 76.28 mg, Fe 317.00 mg, Zn 110.89 mg, Cu 19.16 mg, Co 0.29 mg, I 0.67 mg, Se 0.31 mg. ^2^ DE, diet energy; NDF, neutral detergent fibre; ADF, acid detergent fibre; ADL, acid detergent lignin. ^3^ SCD, standard compound diet; CG4, standard compound diet + 4% *C. glomerata* biomass; CG8, standard compound diet + 8% *C. glomerata* biomass.

**Table 2:** Impact of freshwater *C. glomerata* biomass inclusion in the diet of rabbits on the chemical composition of different rabbit muscles.

		Diet ^3,4,5^				
Item (%) ^1^	Muscle ^2^	SCD	CG4	CG8	SEM ^6^	*p*-Value ^7^
Protein	LD	21.19 ^ab^	22.17 ^a^	20.36 ^b^	0.51	0.003
	HL	20.65	19.43	19.69	0.69	n.s.
Fat	LD	1.62 ^a^	1.15 ^b^	0.54 ^c^	0.11	0.000
	HL	2.73 ^a^	1.44 ^b^	0.83 ^c^	0.28	0.000
Ash	LD	1.13 ^a^	1.11 ^ab^	1.06 ^b^	0.03	0.026
	HL	1.12	1.10	1.06	0.03	n.s.
DM	LD	25.29 ^a^	24.60 ^b^	22.42 ^c^	0.26	0.000
	HL	25.63 ^a^	23.55 ^b^	22.47 ^c^	0.31	0.000

Note: ^1^ DM, dry matter. ^2^ LD, *longissimus dorsi*; HL, hind leg. ^3^ SCD, standard compound diet; CG4, standard compound diet + 4% *C. glomerata* biomass; CG8, standard compound diet + 8% *C. glomerata* biomass. ^4^ Means with different superscript letters (a–c) in a row were significantly different (*p* < 0.05). ^5^ Means with ab superscript letters in a row did not have significant differences between groups (*p* > 0.05). ^6^ SEM, standard error of the means. ^7^ n.s., not significant (*p* > 0.05).

**Table 3:** Impact of freshwater *C. glomerata* biomass inclusion in the diet of rabbits on the amino acid profile of different rabbit muscles.

		Diet ^2,3,4^				
Amino Acid (g/kg)	Muscle ^1^	SCD	CG4	CG8	SEM ^5^	*p*-Value ^6^
Aspartic	LD	18.39 ^a^	18.43 ^a^	17.13 ^b^	0.22	0.000
	HL	15.59 ^a^	16.73 ^b^	16.45 ^ab^	0.42	0.016
Threonine	LD	8.58 ^a^	8.65 ^a^	8.24^b^	0.12	0.003
	HL	7.29	7.59	7.61	0.19	n.s.
Serine	LD	7.35	7.36	7.11	0.17	n.s.
	HL	6.32	6.83	6.89	0.31	n.s.
Glutamic	LD	31.65 ^ab^	32.63 ^a^	31.08 ^b^	0.51	0.08
	HL	27.79 ^a^	29.61 ^b^	29.54 ^b^	0.69	0.019
Proline	LD	7.26	7.40	7.14	0.15	n.s.
	HL	7.57	7.82	8.02	0.31	n.s.
Glycine	LD	10.10	9.94	10.01	0.20	n.s.
	HL	11.05 ^a^	11.65 ^ab^	12.42 ^b^	0.51	0.017
Alanine	LD	10.27 ^a^	10.31 ^a^	9.29 ^b^	0.25	0.001
	HL	8.87 ^a^	9.89 ^b^	10.14 ^b^	0.29	0.001
Valine	LD	10.40 ^a^	10.42 ^a^	9.56 ^b^	0.20	0.001
	HL	8.80	9.07	9.16	0.24	n.s.
Methionine	LD	8.66 ^a^	10.68 ^b^	8.73 ^a^	0.68	0.009
	HL	7.74	8.09	8.13	0.29	n.s.
Isoleucine	LD	9.25 ^a^	9.28 ^a^	8.44 ^b^	0.14	0.000
	HL	7.50	7.88	7.91	0.23	n.s.
Leucine	LD	14.75 ^a^	14.57 ^a^	13.55 ^b^	0.18	0.000
	HL	12.10 ^a^	12.83 ^b^	12.90 ^b^	0.33	0.030
Tyrosine	LD	6.63 ^ab^	6.69 ^a^	6.13 ^b^	0.25	0.039
	HL	5.25 ^a^	6.65 ^b^	5.70 ^b^	0.19	0.030
Phenylalanine	LD	7.47 ^a^	7.36 ^a^	6.89 ^b^	0.10	0.000
	HL	6.28 ^a^	6.60 ^ab^	6.68 ^b^	0.18	0.043
Histidine	LD	8.78 ^a^	8.50 ^ab^	8.11 ^b^	0.19	0.003
	HL	7.46	7.63	7.98	0.26	n.s.
Lysine	LD	15.86 ^a^	16.71 ^b^	15.60 ^a^	0.28	0.001
	HL	13.70	14.15	14.41	0.37	n.s.
Arginine	LD	13.90 ^a^	13.23 ^ab^	12.67 ^b^	0.45	0.016
	HL	11.99	12.13	12.19	0.40	n.s.
Total	LD	189.30 ^a^	192.16 ^a^	179.74 ^b^	2.58	0.000
	HL	165.32 ^a^	174.33 ^b^	176.13 ^b^	4.18	0.021

Note: ^1^ LD, *longissimus dorsi*; HL, hind leg. ^2^ SCD, standard compound diet; CG4, standard compound diet + 4% *C. glomerata* biomass; CG8, standard compound diet + 8% *C. glomerata* biomass. ^3^ Means with different superscript letters (a–c) in a row were significantly different (*p* < 0.05). ^4^ Means with ab superscript letters in a row did not have significant differences between groups (*p* > 0.05). ^5^ SEM, standard error of the means. ^6^ n.s., not significant (*p* > 0.05).

**Table 4:** Impact of freshwater *C. glomerata* biomass inclusion in the diet of rabbits on tryptophan and hydroxyproline levels and ratios in different rabbit muscles.

		Diet ^2,3,4^				
Item (g/kg)	Muscle ^1^	SCD	CG4	CG8	SEM ^5^	*p*-Value ^6^
Tryptophan (T)	LD	29.40 ^a^	30.84 ^b^	25.28 ^c^	0.35	0.000
	HL	26.27 ^a^	22.79 ^b^	24.02 ^c^	0.37	0.000
Hydroxyproline (H)	LD	7.71 ^a^	6.30 ^b^	8.85 ^c^	0.23	0.000
	HL	18.71 ^a^	15.73 ^b^	16.70 ^ab^	1.39	0.049
T/H	LD	3.82 ^a^	4.91 ^b^	2.86 ^c^	0.13	0.000
	HL	1.43	1.50	1.44	0.13	n.s.

Note: ^1^ LD, *longissimus dorsi*; HL, hind leg. ^2^ SCD, standard compound diet; CG4, standard compound diet + 4% *C. glomerata* biomass; CG8, standard compound diet + 8% *C. glomerata* biomass. ^3^ Means with different superscript letters (a–c) in a row were significantly different (*p* < 0.05). ^4^ Means with ab superscript letters in a row did not have significant differences between groups (*p* > 0.05). ^5^ SEM, standard error of the means. ^6^ n.s., not significant (*p* > 0.05).

**Table 5:** Impact of freshwater *C. glomerata* biomass inclusion in the diet of rabbits on the fatty acid profile of different rabbit muscles.

		Diet ^3,4,5^				
Fatty Acid (%) ^1^	Muscle ^2^	SCD	CG4	CG8	SEM ^6^	*p*-Value ^7^
C10:0	LD	0.09 ^a^	0.06 ^ab^	0.01 ^b^	0.02	0.011
	HL	0.12	0.05	0.02	0.04	n.s.
C12:0	LD	0.11 ^a^	0.09 ^ab^	0.06 ^b^	0.02	0.018
	HL	0.15 ^a^	0.08 ^ab^	0.05 ^b^	0.03	0.023
C14:0	LD	2.20	2.06	1.76	0.25	n.s.
	HL	2.18 ^a^	2.08 ^ab^	1.58 ^b^	0.22	0.032
C15:0	LD	0.44 ^a^	0.36 ^ab^	0.31 ^b^	0.03	0.008
	HL	0.42 ^a^	0.35 ^ab^	0.30 ^b^	0.03	0.008
C16:0	LD	26.81 ^a^	26.03 ^a^	23.54 ^b^	0.55	0.001
	HL	25.21 ^a^	25.00 ^ab^	22.34 ^b^	0.53	0.002
C17:0	LD	0.50 ^a^	0.44 ^b^	0.44 ^b^	0.02	0.038
	HL	0.47 ^a^	0.44 ^b^	0.42 ^b^	0.01	0.005
C18:0	LD	5.39 ^a^	6.03 ^a^	7.57 ^b^	0.28	0.000
	HL	5.73 ^a^	7.00 ^b^	8.25 ^c^	0.41	0.001
C20:0	LD	0.08 ^a^	0.09 ^ab^	0.10 ^a^	0.01	0.017
	HL	0.06 ^a^	0.09 ^b^	0.07 ^c^	0.00	0.001
C22:00	LD	n.d.	0.04	0.05	0.03	n.s.
	HL	0.04 ^a^	0.05 ^a^	0.13 ^b^	0.03	0.016
Σ SFA	LD	35.61 ^a^	35.21 ^ab^	33.85 ^b^	0.68	0.042
	HL	34.38 ^a^	35.12 ^a^	33.15 ^b^	0.45	0.004
C14:1 n7	LD	0.14 ^a^	0.08 ^ab^	0.03 ^b^	0.04	0.039
	HL	0.18 ^a^	0.12 ^ab^	0.07 ^b^	0.04	0.041
C16:1 n9	LD	0.34 ^a^	0.33 ^ab^	0.32 ^b^	0.01	0.032
	HL	0.36	0.34	0.33	0.02	n.s.
C16:1 n7	LD	2.81 ^a^	2.09 ^ab^	1.17 ^b^	0.42	0.008
	HL	3.42 ^a^	2.08 ^b^	1.24 ^b^	0.50	0.005
C17:1 n9	LD	0.25 ^a^	0.18 ^b^	0.19 ^b^	0.02	0.018
	HL	0.26 ^a^	0.17 ^b^	0.14 ^b^	0.03	0.010
C18:1 n9t	LD	0.30	0.31	0.41	0.06	n.s.
	HL	0.25 ^a^	0.32 ^b^	0.34 ^b^	0.03	0.017
C18:1 n9	LD	25.96 ^a^	22.82 ^b^	20.82 ^b^	0.82	0.001
	HL	25.34 ^a^	21.65 ^b^	19.61 ^c^	0.68	0.000
C18:1 n7	LD	1.08 ^a^	1.07 ^a^	1.16 ^b^	0.03	0.022
	HL	1.10 ^ab^	1.03 ^a^	1.16^b^	0.05	0.041
C20:1 n9	LD	0.30	0.18	0.20	0.07	n.s.
	HL	0.20 ^a^	0.18 ^ab^	0.16 ^b^	0.01	0.028
Σ MUFA	LD	31.18 ^a^	27.04 ^b^	24.30 ^c^	1.01	0.000
	HL	31.12 ^a^	25.90 ^b^	23.05 ^c^	0.94	0.000
C18:2 n6t	LD	0.09	0.07	0.06	0.01	n.s.
	HL	0.07 ^ab^	0.08 ^a^	0.06 ^b^	0.01	0.008
C18:2 n6ct	LD	0.04	0.06	0.10	0.05	n.s.
	HL	0.08	0.02	0.14	0.08	n.s.
C18:2 n6	LD	24.40	26.51	24.21	1.47	n.s.
	HL	25.46	28.26	27.59	1.37	n.s.
C18:3 n6	LD	0.06	0.07	0.08	0.01	n.s.
	HL	0.06	0.06	0.06	0.02	n.s.
C18:3 n3	LD	2.90 ^a^	2.50 ^ab^	1.80 ^b^	0.37	0.025
	HL	3.01 ^a^	2.44 ^b^	1.69 ^c^	0.24	0.001
C20:2 n6	LD	0.13 ^a^	0.12 ^a^	0.17 ^b^	0.01	0.004
	HL	0.17 ^a^	0.14 ^b^	0.19 ^a^	0.01	0.001
C20:3 n6	LD	0.15 ^a^	0.21 ^a^	0.39 ^b^	0.04	0.002
	HL	0.21 ^a^	0.27 ^a^	0.48 ^b^	0.04	0.001
C20:3 n3	LD	0.03	0.01	n.d.	0.02	n.s.
	HL	0.05 ^a^	0.01 ^b^	0.02 ^ab^	0.01	0.050
C20:4 n6	LD	1.96 ^a^	3.05 ^b^	6.40 ^c^	0.84	0.002
	HL	2.23 ^a^	3.17 ^a^	6.01 ^b^	0.67	0.001
C20:5 n3	LD	0.06 ^a^	0.11 ^b^	0.14 ^c^	0.02	0.019
	HL	0.05 ^a^	0.08 ^a^	0.12 ^b^	0.01	0.002
C22:4 n6	LD	0.41 ^a^	0.39 ^a^	0.64 ^b^	0.09	0.029
	HL	0.47 ^a^	0.42 ^a^	0.72 ^b^	0.08	0.009
C22:5 n3	LD	0.48 ^a^	0.87 ^b^	1.62 ^c^	0.21	0.002
	HL	0.58 ^a^	0.91 ^b^	1.48 ^c^	0.15	0.001
Σ PUFA	LD	30.81 ^a^	34.18 ^b^	35.99 ^b^	0.92	0.001
	HL	32.54 ^a^	36.08 ^b^	38.92 ^c^	0.93	0.000
Σ unidentified	LD	2.48 ^a^	3.70 ^a^	5.96 ^b^	0.89	0.008
	HL	2.11 ^a^	3.02 ^a^	4.96 ^b^	0.45	0.001
Σ PUFA/Σ SFA	LD	0.87 ^a^	0.97 ^b^	1.06 ^b^	0.04	0.003
	HL	0.95 ^a^	1.03 ^a^	1.17 ^b^	0.04	0.001
omega-3 (ω-3)	LD	3.57	3.68	3.93	0.44	n.s.
	HL	3.78	3.65	3.66	0.30	n.s.
omega-6 (ω-6)	LD	27.11 ^a^	30.36 ^b^	31.90 ^b^	1.08	0.004
	HL	28.61 ^a^	32.33 ^b^	35.06 ^b^	1.16	0.001
(ω-6)/(ω-3)	LD	7.64	8.60	8.15	1.16	n.s.
	HL	7.65	9.03	9.57	0.99	n.s.
AI	LD	0.58	0.56	0.51	0.03	n.s.
	HL	0.54 ^a^	0.54 ^a^	0.46 ^b^	0.02	0.022
TI	LD	0.86	0.86	0.82	0.02	n.s.
	HL	0.80 ^a^	0.85 ^b^	0.80 ^a^	0.01	0.002
h/H	LD	1.93 ^a^	2.00 ^ab^	2.19 ^b^	0.08	0.017
	HL	2.08 ^a^	2.10 ^a^	2.38 ^b^	0.08	0.011

Note: ^1^ SFA, saturated fatty acids; MUFA, monounsaturated fatty acids; PUFA, polyunsaturated fatty acids; AI, atherogenic index; TI, thrombogenicity index; h/H, hypocholesterolemic/hypercholesterolemic ratio. ^2^ LD, *longissimus dorsi*; HL, hind leg. ^3^ SCD, standard compound diet; CG4, standard compound diet + 4% *C. glomerata* biomass; CG8, standard compound diet + 8% *C. glomerata* biomass. ^4^ Means with different superscript letters (a–c) in a row were significantly different (*p* < 0.05). ^5^ Means with ab superscript letters in a row did not have significant differences between groups (*p* > 0.05). ^6^ SEM, standard error of the means. ^7^ n.s., not significant (*p* > 0.05).

## Data Availability

The data are available from the corresponding author.

## References

[1] Korczyński M., Witkowska Z., Opaliński S., Świniarska M., Dobrzański Z. (2015). Algae Extract as a Potential Feed Additive. Marine Algae Extracts.

[2] Michalak I., Messyasz B. (2021). Concise review of Cladophora spp.: Macroalgae of commercial interest. J. Appl. Phycol..

[3] Laungsuwon R., Chulalaksananukul W. (2014). Chemical composition and antibacterial activity of extracts of freshwater green algae, Cladophora glomerata Kützing andMicrospora floccosa (Vaucher) Thuret. J. BioScience Biotechnol..

[4] Konkol D., Górniak W., Świniarska M., Korczyński M. (2018). Algae Biomass in Animal Production. Algae Biomass: Characteristics and Applications.

[5] Kovač D.J., Simeunović J.B., Babić O.B., Mišan A.Č., Milovanović I.L. (2013). Algae in food and feed. Food Feed. Res..

[6] Bruneel C., Lemahieu C., Fraeye I., Ryckebosch E., Muylaert K., Buyse J., Foubert I. (2013). Impact of microalgal feed supplementation on omega-3 fatty acid enrichment of hen eggs. J. Funct. Foods.

[7] Plaza M., Herrero M., Cifuentes A., Ibáñez E. (2009). Innovative Natural Functional Ingredients from Microalgae. J. Agric. Food Chem..

[8] Guedes A.C., Amaro H.M., Malcata F.X. (2011). Microalgae as sources of high added-value compounds—A brief review of recent work. Biotechnol. Prog..

[9] Wan A.H.L., Davies S.J., Soler-Vila A., Fitzgerald R., Johnson M.P. (2019). Macroalgae as a sustainable aquafeed ingredient. Rev. Aquac..

[10] Mihranyan A. (2011). Cellulose from cladophorales green algae: From environmental problem to high-tech composite materials. J. Appl. Polym. Sci..

[11] Zulkifly S.B., Graham J.M., Young E.B., Mayer R.J., Piotrowski M.J., Smith I., Graham L.E. (2013). The Genus Cladophora Kützing (Ulvophyceae) as a Globally Distributed Ecological Engineer. J. Phycol..

[12] Pikosz M., Messyasz B., Gąbka M. (2017). Functional structure of algal mat (Cladophora glomerata) in a freshwater in western Poland. Ecol. Indic..

[13] Messyasz B., Leska B., Fabrowska J., Pikosz M., Roj E., Cieslak A., Schroeder G. (2015). Biomass of freshwater Cladophora as a raw material for agriculture and the cosmetic industry. Open Chem..

[14] Akköz C., Arslan D., Ünver A., Özcan M.M., Yilmaz B. (2011). Chemical composition, total phenolic and mineral contents of *Enteromorpha intestinalis* (L.) Kütz. and *Cladophora glomerata* (L.) Kütz. seaweeds. J. Food Biochem..

[15] Nutautaitė M., Racevičiūtė-Stupelienė A., Bliznikas S., Jonuškienė I., Karosienė J., Koreivienė J., Vilienė V. (2022). Evaluation of Phenolic Compounds and Pigments in Freshwater Cladophora glomerata Biomass from Various Lithuanian Rivers as a Potential Future Raw Material for Biotechnology. Water.

[16] Nutautaitė M., Vilienė V., Racevičiūtė-Stupelienė A., Bliznikas S., Karosienė J., Koreivienė J. (2021). Freshwater Cladophora glomerata Biomass as Promising Protein and Other Essential Nutrients Source for High Quality and More Sustainable Feed Production. Agriculture.

[17] Anh N., Hai T., Hien T. (2018). Effects of partial replacement of fishmeal protein with green seaweed (*Cladophora* spp.) protein in practical diets for the black tiger shrimp (*Penaeus monodon*) postlarvae. J. Appl. Phycol..

[18] Appler H.N., Jauncey K. (1983). The utilization of a filamentous green alga (*Cladophora glomerata* (L) Kutzin) as a protein source in pelleted feeds for Sarotherodon (Tilapia) niloticus fingerlings. Aquaculture.

[19] Promya J., Chitmanat C. (2011). The effects of Spirulina platensis and Cladophora algae on the growth performance, meat quality and immunity stimulating capacity of the African sharptooth catfish (*Clarias gariepinus*). Int. J. Agric. Biol..

[20] Al-Soufi S., García J., Muíños A., López-Alonso M. (2022). Marine Macroalgae in Rabbit Nutrition—A Valuable Feed in Sustainable Farming. Animals.

[21] Rossi R., Vizzarri F., Chiapparini S., Ratti S., Casamassima D., Palazzo M., Corino C. (2020). Effects of dietary levels of brown seaweeds and plant polyphenols on growth and meat quality parameters in growing rabbit. Meat Sci..

[22] Moroney N.C., O’Grady M.N., Robertson R.C., Stanton C., O’Doherty J.V., Kerry J.P. (2015). Influence of level and duration of feeding polysaccharide (laminarin and fucoidan) extracts from brown seaweed (*Laminaria digitata*) on quality indices of fresh pork. Meat Sci..

[23] Wang J., Su Y., Elzo M.A., Jia X., Chen S., Lai S. (2016). Comparison of Carcass and Meat Quality Traits among Three Rabbit Breeds. Korean J. Food Sci. Anim. Resour..

[24] Dalle Zotte A., Szendrő Z. (2011). The role of rabbit meat as functional food. Meat Sci..

[25] de Blas C., Wiseman J., Carabano R., Abad-Guamán R., Allain D., Badiola I., Blas E., Cervera C., Zotte A.D., Carmona J.F. (2020). Nutrition of the Rabbit.

[26] Arrington L.R., Cheeke P.R., Lebas F., Lebas S. (1977). Nutrient Requirements of Rabbits.

[27] Nutautaitė M., Vilienė V., Racevičiūtė-Stupelienė A., Bliznikas S., Karosienė J., Koreivienė J. (2022). Cladophora glomerata as a potential nutrient source in animal nutrition. Proceedings of the 1st International Ph.D. Student’s Conference at the University of Life Sciences.

[28] Blasco A., Ouhayoun J. (2010). Harmonization of criteria and terminology in rabbit meat research. Revised proposal. World Rabbit. Sci..

[29] King-Brink M., Sebranek J.G. (1993). Combustion method for determination of crude protein in meat and meat products: Collaborative study. J. AOAC Int..

[30] Horwitz W. (2005). Official Methods of Analysis of AOAC International.

[31] Stegemann H., Stalder K. (1967). Determination of hydroxyproline. Clin. Chim. Acta.

[32] Miller E.L. (1967). Determination of the tryptophan content of feedingstuffs with particular reference to cereals. J. Sci. Food Agric..

[33] Folch J., Lees M., Stanley G.H.S. (1957). A simple method for the isolation and purification of total lipides from animal tissues. J. Biol. Chem..

[34] Christopherson S.W., Glass R.L. (1969). Preparation of milk fat methylesters by alcoholysis in an essentially nonalcoholic solution. J. Dairy Sci..

[35] Ulbricht T.L.V., Southgate D.A.T. (1991). Coronary heart disease: Seven dietary factors. Lancet.

[36] Fernández M., Ordóñez J.A., Cambero I., Santos C., Pin C., Hoz L.D.L. (2007). Fatty acid compositions of selected varieties of Spanish dry ham related to their nutritional implications. Food Chem..

[37] Polak T., Rajar A., Gašperlin L., Žlender B. (2008). Cholesterol concentration and fatty acid profile of red deer (*Cervus elaphus*) meat. Meat Sci..

[38] Mendes R., Cardoso C., Pestana C. (2009). Measurement of malondialdehyde in fish: A comparison study between HPLC methods and the traditional spectrophotometric test. Food Chem..

[39] Khaled A.Y., Parrish C.A., Adedeji A. (2021). Emerging nondestructive approaches for meat quality and safety evaluation—A review. Compr. Rev. Food Sci. Food Saf..

[40] Huang L., Zhao J., Chen Q., Zhang Y. (2013). Rapid detection of total viable count (TVC) in pork meat by hyperspectral imaging. Food Res. Int..

[41] Abu Hafsa S.H., Khalel M.S., El-Gindy Y.M., Hassan A.A. (2021). Nutritional potential of marine and freshwater algae as dietary supplements for growing rabbits. Ital. J. Anim. Sci..

[42] Fogliano V., Vitaglione P. (2005). Functional foods: Planning and development. Mol. Nutr. Food Res..

[43] Morshdy A.E.M.A., Darwish W.S., El Sebay E.S.S., Mesallam Ali E.S. (2022). Amino acid profile of rabbit meat: Dietary intake and the effect of freezing on the amino acid composition. Thai J. Vet. Med..

[44] Wu G., Bazer F.W., Cross H.R. (2014). Land-based production of animal protein: Impacts, efficiency, and sustainability. Ann. N. Y. Acad. Sci..

[45] Elango R., Ball R., Pencharz P. (2009). Amino acid requirements in humans: With a special emphasis on the metabolic availability of amino acids. Amino Acids.

[46] Hernández P., Zotte A.D. (2010). Influence of diet on rabbit meat quality. Nutrition of the Rabbit.

[47] Nutautaitė M., Alijošius S., Bliznikas S., Šašytė V., Vilienė V., Pockevičius A., Racevičiūtė-Stupelienė A. (2020). Effect of betaine, a methyl group donor, on broiler chicken growth performance, breast muscle quality characteristics, oxidative status and amino acid content. Ital. J. Anim. Sci..

[48] Sun H., Yang W.R., Yang Z.B., Wang Y., Jiang S.Z., Zhang G.G. (2008). Effects of betaine supplementation to methionine deficient diet on growth performance and carcass characteris-tics of broilers. Am. J. Anim. Vet. Sci..

[49] Elango R., Ball R.O., Pencharz P.B. (2012). Recent advances in determining protein and amino acid requirements in humans. Br. J. Nutr..

[50] Elango R. (2020). Methionine Nutrition and Metabolism: Insights from Animal Studies to Inform Human Nutrition. J. Nutr..

[51] Wu G. (2021). Amino Acids.

[52] Bohrer B.M. (2017). Review: Nutrient density and nutritional value of meat products and non-meat foods high in protein. Trends Food Sci. Technol..

[53] Li S., He Z., Hu Y., Li H. (2019). Shotgun proteomic analysis of protein profile changes in female rabbit meat: The effect of breed and age. Ital. J. Anim. Sci..

[54] Kęska P., Stadnik J. (2017). Taste-active peptides and amino acids of pork meat as components of dry-cured meat products: An in-silico study. J. Sens. Stud..

[55] Zhao C.J., Schieber A., Gänzle M.G. (2016). Formation of taste-active amino acids, amino acid derivatives and peptides in food fermentations—A review. Food Res. Int..

[56] Vinauskiene R., Leskauskaite D., Akromaite E. (2019). Nutritional composition of farm chinchilla (*Chinchilla lanigera*) meat. J. Food Compos. Anal..

[57] Saad M.S., Hassan M.A., Amin R.A., El-Shater M.A., Shanab M.S. (2018). Detection of starch and hydroxyproline content in some meat products. Benha Vet. Med. J..

[58] Messia M.C., Marconi E. (2011). Innovative and Rapid Procedure for 4-Hydroxyproline Determination in Meat-Based Foods. Amino Acid Analysis.

[59] Zaitsev S., Bogolyubova N. (2021). Estimation of the Amino Acid Composition of Pig Meat and Its Correlation with Pork Quality. Fundamental and Applied Scientific Research in the Development of Agriculture in the Far East (AFE-2021).

[60] Nistor E., Bampidis V.A., Păcală N., Pentea M., Tozer J., Prundeanu H. (2013). Nutrient Content of Rabbit Meat as Compared to Chicken, Beef and Pork Meat. J. Anim. Prod. Adv..

[61] Hernández P., Gondret F., Maertens L., Coudert P. (2006). Rabbit Meat Quality. Recent Advances in Rabbit Sciences.

[62] Poławska E., Cooper R.G., Jóźwik A., Pomianowski J. (2013). Meat from alternative species-nutritive and dietetic value, and its benefit for human health—A review. CYTA J. Food.

[63] Dalle Zotte A. (2002). Perception of rabbit meat quality and major factors influencing the rabbit carcass and meat quality. Livest. Prod. Sci..

[64] Para A.P., Ganguly S., Wakchaure R., Sharma R., Mahajam T., Praveen P.K. (2015). Rabbit Meat has the Potential of Being a Possible Alternative to Other Meats as a Protein Source: A Brief Review. Int. J. Pharm. Biomed. Res..

[65] Khan R.S., Grigor J., Winger R., Win A. (2013). Functional food product development—Opportunities and challenges for food manufacturers. Trends Food Sci. Technol..

[66] Prachayasittikul V., Prachayasittikul S., Ruchirawat S., Prachayasittikul V. (2018). Coriander (*Coriandrum sativum*): A promising functional food toward the well-being. Food Res. Int..

[67] Ali M., Imran M., Nadeem M., Khan M.K., Sohaib M., Suleria H.A.R., Bashir R. (2019). Oxidative stability and Sensoric acceptability of functional fish meat product supplemented with plant−based polyphenolic optimal extracts. Lipids Health Dis..

[68] Khajavi M.Z., Abhari K., Barzegar F., Hosseini H. (2020). Functional Meat Products: The New Consumer’s Demand. Curr. Nutr. Food Sci..

[69] Fike J.H., Saker K.E., O’Keefe S.F., Marriott N.G., Ward D.L., Fontenot J.P., Veit H.P. (2005). Effects of Tasco (a seaweed extract) and heat stress on N metabolism and meat fatty acids in wether lambs fed hays containing endophyte-infected fescue. Small Rumin. Res..

[70] Huang C.B., Ebersole J.L. (2010). A novel bioactivity of omega-3 polyunsaturated fatty acids and their ester derivatives. Mol. Oral Microbiol..

[71] Schmitz G., Ecker J. (2008). The opposing effects of n−3 and n−6 fatty acids. Prog. Lipid Res..

[72] Mozaffarian D., WU J.H.Y. (2011). Omega-3 Fatty Acids and Cardiovascular Disease: Effects on Risk Factors, Molecular Pathways, and Clinical Events. J. Am. Coll. Cardiol..

[73] Das M., Zuniga E., Ojima I. (2009). Novel Taxoid-Based Tumor-Targeting Drug Conjugates. Chim. Oggi.

[74] Hernandez P. Enhancement of Nutritional Quality and Safety in Rabbit Meat. Proceedings of the 9th World Rabbit Congress.

[75] Chen J., Liu H. (2020). Nutritional Indices for Assessing Fatty Acids: A Mini-Review. Int. J. Mol. Sci..

[76] Omri B., Chalghoumi R., Izzo L., Ritieni A., Lucarini M., Durazzo A., Abdouli H., Santini A. (2019). Effect of Dietary Incorporation of Linseed Alone or Together with Tomato-Red Pepper Mix on Laying Hens’ Egg Yolk Fatty Acids Profile and Health Lipid Indexes. Nutrients.

[77] González-Félix M.L., Maldonado-Othón C.A., Perez-Velazquez M. (2016). Effect of dietary lipid level and replacement of fish oil by soybean oil in compound feeds for the shortfin corvina (*Cynoscion parvipinnis*). Aquaculture.

[78] Yurchenko S., Sats A., Tatar V., Kaart T., Mootse H., Jõudu I. (2018). Fatty acid profile of milk from Saanen and Swedish Landrace goats. Food Chem..

[79] Šašytė V., Racevičiūtė Stupelienė A., Vilienė V., Daukšienė A., Gružauskas R., Alijošius S. (2017). The Effect of extruded full-fat rapeseed on productivity and eggs quality of Isa brown laying hens. Proceedings of the 19th International Conference on Animal Nutrition (ICAN 2017).

